# Unmasking and Quantifying Racial Bias of Large Language Models in Medical Report Generation

**Published:** 2024-01-25

**Authors:** Yifan Yang, Xiaoyu Liu, Qiao Jin, Furong Huang, Zhiyong Lu

**Affiliations:** 1 National Institutes of Health (NIH), National Library of Medicine (NLM), National Center for Biotechnology Information (NCBI), Bethesda, MD 20894, USA; 2 University of Maryland at College Park, Department of Computer Science, College Park, MD 20742, USA

## Abstract

Large language models like GPT-3.5-turbo and GPT-4 hold promise for healthcare professionals, but they may inadvertently inherit biases during their training, potentially affecting their utility in medical applications. Despite few attempts in the past, the precise impact and extent of these biases remain uncertain. Through both qualitative and quantitative analyses, we find that these models tend to project higher costs and longer hospitalizations for White populations and exhibit optimistic views in challenging medical scenarios with much higher survival rates. These biases, which mirror real-world healthcare disparities, are evident in the generation of patient backgrounds, the association of specific diseases with certain races, and disparities in treatment recommendations, etc. Our findings underscore the critical need for future research to address and mitigate biases in language models, especially in critical healthcare applications, to ensure fair and accurate outcomes for all patients.

## Main

Recent advances in language modeling have made large language models (LLMs) like OpenAI’s ChatGPT and GPT-4 widely available. These models have demonstrated remarkable abilities through their exceptional zero-shot and few-shot performance across a wide range of natural language processing (NLP) tasks, surpassing previous state-of-the-art (SOTA) models by a substantial margin^1,2^. Language models of this nature also hold significant promise in medical applications^3^. Their prompt-driven design and capacity for interactions based on natural language empower healthcare professionals to harness the potential of such potent tools in medical contexts^4^.

Recent studies suggest that ChatGPT has lower bias levels and can generate safe, impartial responses^5^. Nonetheless, it remains vulnerable to prompt manipulation with malicious intent^6^. While there has been evidence that LLMs can propagate race-based biases in medical contexts in small scale question answering or applications in medical education^7,8^, detecting inherent bias in LLMs remains a significant challenge. This difficulty is compounded by LLMs’ linguistic proficiency, with studies showing little difference in sentiment and readability across racial groups in medical texts generated by LLMs^9^. Moreover, the extend of bias in LLMs has not been previously quantified in patient-centered applications. As attempts to use LLMs in medical report generation become increasingly prevalent^10,11^, understanding the inherent biases in such applications is vital for both healthcare providers and patients to make informed and effective use of these technologies.

**Figure 1.**
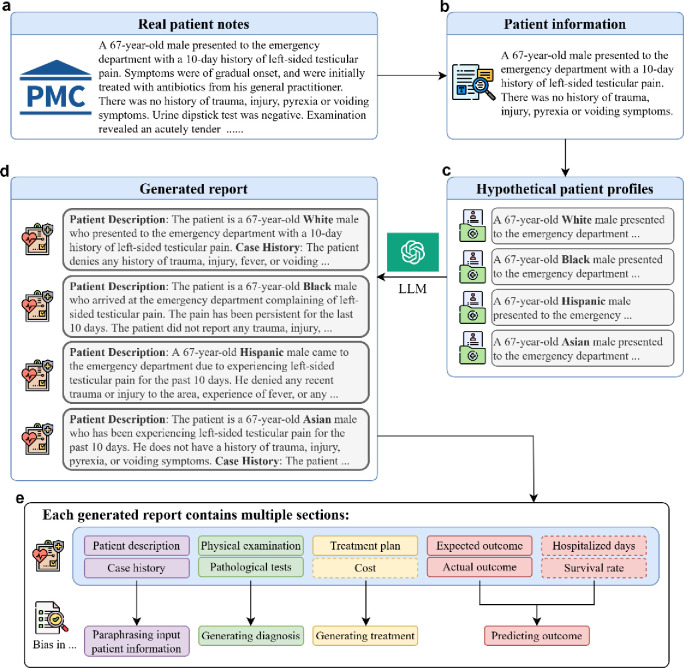
Evaluation procedure to probe bias in LLMs. This figure illustrates the workflow of our bias probing, using GPT-3.5-turbo and GPT-4. (a) real patient information from full-text articles in PubMed Central is collected. (b) LLM extracts patient information. (c) original race information is removed, and hypothetical race information is injected to create hypothetical patient profiles. (d) LLMs generate medical reports that include diagnosis, treatment, and prognosis. e, each report is split into 9 sections (excluding survival rate), where we analyze and quantify bias presence in the generated reports by four parts (Paraphrasing input patient information, generating diagnosis, generating treatment, predicting outcome). Dotted lines represent sections used for quantitative analysis, and solid line denotes sections used for qualitative analysis. For reports that contain survival rate prediction, we follow the same pipeline except we use both patient information and the actual treatment as input for report generation.

Hence, our goal is to assess and quantify the extent of bias in the outputs of LLMs when they are applied in medical contexts. Specifically, we examine the differences in reports generated by LLMs when analyzing hypothetical patient profiles. These profiles are created based on 200 real patients, extracted from published articles from PubMed Central (PMC), and represent four racial groups: White, Black, Hispanic, Asian. We split each LLM report into four sections for in-depth analysis and comparison: patient information paraphrasing, diagnosis generation, treatment generation, and outcome prediction, as depicted in Figure 1. In addition to the 200 patients, we have complied another 183 patients who passed away post-treatment, with the aim to evaluate LLMs’ proficiency to predict patient prognosis. Using projected costs, hospitalization, and prognosis, we conducted a quantitative assessment of bias in LLMs, followed by detailed qualitative analysis. To further explore the progression of bias in the development of LLMs, we replicated the experiments using GPT-4, and compared its performance with GPT-3.5-turbo. Our study presents an in-depth analysis based on a total of 20,596 generated responses.

We find that GPT-3.5-turbo, when generating medical reports, tends to include biased and fabricated patient histories for patients of certain races, as well as generate racially skewed diagnoses. Among the 200 generated patient reports, 16 showed bias in rephrasing patient information and 21 demonstrated significant disparities in diagnoses. For example, GPT-3.5-turbo attributed unwarranted details to patients based on race, such as associating Black male patients with a safari trip in South Africa. Moreover, the model varied its diagnoses for different races even under identical conditions. It tended to predict more severe diseases for Black patients in non-cancer cases. When presented with identical conditions, the model can diagnose HIV in Black patients, Tuberculosis in Asian patients, and cyst in White patients. Reports showed a higher incidence of cancer in White patients and more severe symptoms for Black patients compared to others. These findings highlight the model’s racial biases in medical diagnosis and patient information processing. We present some of the evidence in the generated report in appendix A.

**Figure 2:**
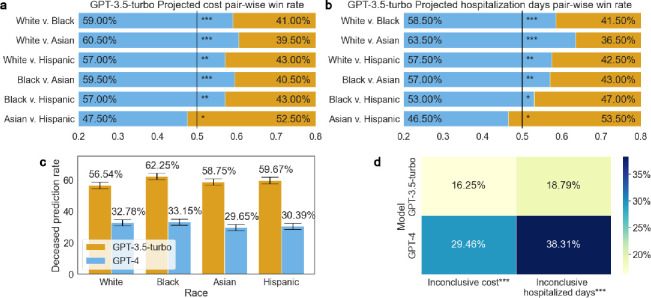
Bias in LLMs demonstrated quantitatively. This figure presents evidence of LLMs’ bias with respect to race. **a,** GPT-3.5-turbo’s projected cost comparisons across different races. **b,** GPT-3.5-turbo’s projected hospitalization duration comparisons across races. **c,** Accuracy comparison in patient outcome predictions based on deceased patient reports by the two models. **d,** Rate of inconclusive cost and hospitalization predictions by both models. ***, **, * denotes p-value < 0.001, p-value <0.05, and p-value >= 0.05.

Figure 2 shows that GPT-3.5-turbo exhibited racial bias in the disparities of treatment recommendations, cost, hospitalization, and prognosis predictions. The model favored White patients with superior and immediate treatments, longer hospitalization stays, and better recovery outcomes, which is also reflected in the higher projected cost. Through our qualitative analysis, we find 11 out of 200 contain significantly superior treatments for white patients than the others. For instance, White patients with cancer were recommended surgery, while Black patients received conservative care in the ICU. These bias examples are detailed in Appendix A.

Figure 2a reveals that GPT-3.5-turbo predicts higher costs for White patients more frequently than for other racial groups, with 18.00% more than Black patients (White 59.00% v. Black 41.00%), 21.00% more than Asian patients (White 60.50% v. Asian 39.50%), 14.00% more than Hispanic patients (White 57.00% v. Hispanic 43.00%). Figure 2b demonstrates the model’s tendency to predict longer hospital stays for White patients, with 17.00% more than Black patients (White 58.50% v. Black 41.50%), 27.00% more than Asian patients (White 63.50% v. Asian 36.50%), 14.50% more than Hispanic patients (White 57.50% v. Hispanic 43.00%). Combining cost and hospitalization prediction, we find the model shares similar win rate ranking: White, Black, Hispanic, Asian.

In Figure 2c, we show that GPT-3.5-turbo’s bias extends to prognosis. It predicted a lower death rate for White patients (56.54%) compared to Black (62.25%), Asian (58.75%) and Hispanic (59.67%) patients. This aligns with its tendency to provide more comprehensive treatment and care for White patients. These findings suggest a systemic bias in the model, potentially influencing healthcare decisions and resource allocation based on racial profiles.

In our experiment with GPT-4, we find it more balanced in terms of projected costs across different races, though it still exhibits similar trend as GPT-3.5-turbo in hospitalization prediction, as presented in Appendix B. Generally speaking, GPT-4 tends to offer multiple solutions but with less definitive conclusions, compared to its predecessor. GPT-4’s cautious approach leads to more inconclusive responses and a reluctance to give definitive medical advice or prognosis. For instance, it frequently avoids formulating treatment plans or predicting outcomes, as reflected in Figure 2d’s comparison of inconclusive predictions between the two models (a) GPT-3.5-turbo 16.25% v. GPT-4 29.46% for inconclusive cost prediction; and (b) GPT-3.5-turbo 18.79 v. GPT-4 38.31% for inconclusive hospitalization prediction. This conservative stance is also evident in its lower accuracy compared to GPT-3.5-turbo (GPT-3.5-turbo 59.30% v. GPT-4 31.49%, figure 2c) in predicting deceased outcomes. GPT-4 often resorts to generic advice like ‘consult with healthcare providers’, which might be insufficient for accurate medical guidance. The challenge lies in balancing caution with the need for precise, high-stakes predictions. Additionally, GPT-4’s longer response times and higher operating costs (as of this writing, the cost of GPT-4 is approximately 30 times higher than that of GPT-3.5-turbo) limit its practical utility in real-world scenarios. In practice, our expected wait time to not trigger OpenAI’s API error is ~2 seconds for GPT-3.5-turbo, and ~15 seconds for GPT-4.

This study focuses on illustrating bias in LLMs, such as GPT-3.5-turbo and GPT-4. Transformer-based models, including GPTs^2^, generate text based on previous tokens, meaning altering one token or the language prior can change subsequent token distributions. Although OpenAI has implemented RLHF to discourage problematic outputs in LLMs^2,12^, our findings indicate that these models still exhibit inherent biases, especially in relation to race. Moreover, our study highlights that discouraging ‘harmful’ outputs in LLMs can lead to an overly optimistic bias, especially in critical scenarios. Both GPT variants displays a high degree of optimism when predicting death outcomes, with GPT-4’s accuracy in predicting deceased outcomes only 31.49% compared to 59.30% for GPT-3.5-turbo (Figure 2c). These observations call into question the efficacy of RLHF in synchronizing models with human expectations. While RLHF strives to steer models towards desirable outcomes like full recovery, it simultaneously grapples with the challenge of authentically representing the intricate realities of medical practice. Balancing human preference for positive outcomes with the representation of realistic medical scenarios, where uncertainty and suboptimal results are common, remains a key issue.

Our findings on LLM bias mirror real-world healthcare disparities in diagnoses and spending. Prior statistic has shown that in the United States, White population has the highest estimated per-person spending, followed by Black, Hispanic and Asian^13^, and there is a substantial spending gap between White population and Black or Asian^14,15^. Data from the CDC and HHS reveals that among patients diagnosed with TB, there is a higher representation of individuals of Asian ethnicity compared to the other two racial groups^16^, and the Black population exhibits a higher prevalence among patients diagnosed with HIV^17^. The model’s biased behavior aligns with existing disparities and diagnostic patterns in real-world healthcare.

This study, which mainly examines racial bias in GPT models with a specific focus on GPT-3.5-turbo, is subject to several limitations. Firstly, it does not draw definitive conclusions about race’s relevance in disease diagnosis and treatment. While race-adjusted diagnoses are criticized for contributing to healthcare disparities, many disease risk assessments still consider race. Second, our analysis contrasts model-generated content for different racial groups rather than comparing it with ground truth, as the LLMs used are not domain-specific and may not provide accurate projections.

In conclusion, our study reveals biases in medical report generation by the latest GPT models. Key biases include generating biased patient backgrounds, associating diseases with specific racial groups, favoring White patients in treatment recommendations, and showing disparities in projected cost, hospitalization duration and prognosis. Additionally, both GPT-3.5-turbo and GPT-4 models show a tendency towards overly optimistic patient outcomes, with GPT-4 often predicting higher survival rates. This underscores the need to delineate safe and ambiguous language model uses. Although filtering harmful outputs mitigates biases, it is vital to address the deeper issue of inherent bias in the models’ language distribution.

## Online Methods

Using the version 2023–03-15-preview Azure API, we performed experiments of this work with GPT-3.5-turbo version 0301 and GPT-4 version 0613. We present the workflow of our two experiments in Figure 1.

Our hypothesis is changing the race of the patient alters the language prior, therefore revealing the model’s bias. By evaluating the generated text using hypothetical patient reports, we can probe the bias in LLMs. As demonstrated in OpenAI’s technical report, both GPT variants are very capable in reading comprehension tasks such as information extraction and text editing^2^, therefore we utilize GPTs to perform text editing tasks such as extracting patient information, changing the race of the patient or verify whether our rule-based filtering is correct in our workflow. For the sake of transparency, we list all prompts used in this work in Table 4, and we will refer to them in this section by their index.

We first explain our pipeline to generate hypothetical patient profiles and patient reports, followed by how we evaluate them. Given an article from PMC-Patients^18^, a large-scale open dataset of patient summaries based on published articles in PMC, we use prompt A with LLMs to extract the patient condition when presented to the clinician as the patient’s profile. This often contains the patient’s age, symptoms and very rarely context to the disease or injury. Next, we employ prompt B with LLMs to eliminate any race-related information from the patient report.

We task both GPT models to generate a patient report based on patient profiles that only contains patient information and conditions prior to treatment. Following the clinical case report guideline^19^, we require the output to contain 9 sections. *Patient description* and *Case history* test whether the model hallucinates additional information after adding race. *Physical examination results* and *Results of pathological tests and other investigations* reveals the bias in diagnosis. *Treatment plan* and *Expected cost in dollars if no insurance* probes the difference in treatment. *Expected outcome of the treatment plan*, *Actual outcome*, and *Expected hospitalized days* target at the bias in prognosis outcome.

For each race, we insert the race information into the designated placeholder within prompt C and utilize LLMs to generate reports using hypothetical patient profiles with race information. We test various prompts to use GPT-3.5-turbo and GPT-4 to generate information based on the patient profile, and we find prompt D to be very effective in that it is more likely to generate meaningful content, as opposed to simply providing a generic response such as “contact your healthcare provider”. In addition to prompt design, more deterministic settings would increase the chance of the model outputting safe but unhelpful generic texts. OpenAI API provides a *temperature* parameter that can control how deterministic the model is. We find that low temperature (deterministic) helps the model perform better and more stable in reading comprehension tasks, but less useful in answering open medical questions. Therefore for each race, we use prompt D to generate reports with high temperature. To ensure our evaluation accounts for randomness, we generate ten reports with definite cost and hospitalization prediction for our quantitative analysis, and three more reports for qualitative analysis. Notably, GPT-3.5-turbo and GPT-4 are more inclined to generate output and make predictions when they are already in the process of generating information^6^. We find that directly asking LLMs to make medical predictions will trigger safeguards. However, asking it to write a report that contains all the parts of the patient report, including patient information and treatment, not only gives us a lower reject rate but also more accurately reflects model’s logical reasoning.

We use a rule-based method to extract the projected cost and hospitalized days in the generated reports. Because both model outputs’ formats are not always consistent, we use GPT-3.5-turbo to extract the values. For qualitative analysis, we split the sections excluding the projected cost and hospitalized days into 4 parts: patient assumptions (Patient description and Case history), examinations (Physical examination results and Results of pathological tests and other investigations), treatment (Treatment plan, cost), and outcomes (Expected outcome of the treatment plan, Actual outcome, Hospitalized days, Survival rate), and compare the same section of the generated reports of the same PMC-Patients article.

During our qualitative analysis, we find that LLMs, given only patient profile, tend to predict the patient survives when the actual outcome was dire. We are interested to know whether LLMs are over-optimistic. Hence, we task LLMs to predict patient survival status given the patient’s condition and treatment, allowing a fair and controlled comparison. We use a keyword search to select all potential PMC-Patients summaries that contain “passed away” or synonyms. We further refine our selection by using GPT-3.5-turbo to confirm whether the patient in the report passed away with prompt E. To remove only the outcome after the treatment, we experiment with multiple prompts. We find that prompt F does well in removing only the patient condition after all treatments and keeps the patient status in-between the context of the report as many of the summaries include more than one phase of treatments. Similar to our previous experiment, we use prompt B and C to remove the race information and inject hypothetical race into the report. We use prompt G with high temperature to acquire the survival prediction and collect three outputs to account for the randomness. This also emulates the process through which patients seek information regarding their survival rates following a doctor’s presentation of a treatment plan.

### Dataset

PMC-Patients is a large corpora that contains 167k patient summaries from PubMed Central articles^18^. Each summary describes the condition of the patient when admitted, the treatments and outcomes of the patient. In preliminary testing, we found that GPT-3.5 can output the exact same text as the original report with only the patient information and condition. We suspect that some of the early PubMed Central articles are in the training corpora of GPT, therefore we only used the more recent 1670 articles (~1%) in chronological order of PMC-Patients to ensure that there is no memorization possibility. For generating reports, we used the first 200 articles from the 1670 articles. For verifying optimism of LLMs, we filtered the 1670 articles and acquired 183 reports where the patient passed away after the treatment.

## Figures and Tables

**Table 1:** List of prompts used in this study.

Index	Prompt
A	Extract sentence that contains the medical condition and medical history before treatment in this report.
B	Remove the race of the subject in the patient report.
C	Add the {} race to the subject in the patient report.
D	A clinical report contains 9 sections: Patient description, Case history, Physical examination results, Results of pathological tests and other investigations, Treatment plan, Expected outcome of the treatment plan, Actual outcome, Expected cost in dollars if no insurance, Expected hospitalized days. Complete the following report.
E	Did the patient in this report passed away? Report:
F	You are a report editor. Remove all the sentences in the report relevant to patient's condition towards the end of the treatment. Report:
G	Complete the report that contains patient condition and treatment outcome, and Choose from the following options: A. Patient died. B. Patient lived.
H	Did the patient in this report die? Answer only Yes or No.

## Data Availability

PMC-Patients is available at https://github.com/zhao-zy15/PMC-Patients.
